# Characterization of exercise limitations by evaluating individual cardiac output patterns: a prospective cohort study in patients with chronic heart failure

**DOI:** 10.1186/s12872-015-0057-6

**Published:** 2015-06-23

**Authors:** Ruud F. Spee, Victor M. Niemeijer, Bart Wessels, Jasper P. Jansen, Pieter F.F. Wijn, Pieter A.F.M. Doevendans, Hareld M.C. Kemps

**Affiliations:** Department of Cardiology, Máxima Medical Centre, De Run 4600, P.O. Box 7777, Veldhoven, 5500 MB The Netherlands; Department of Biomedical Engineering, Eindhoven University of Technology, Eindhoven, The Netherlands; Department of Clinical Physics and Clinical Informatics, Máxima Medical Centre Veldhoven, Veldhoven, The Netherlands; Department of Applied Physics, Eindhoven University of Technology, Eindhoven, The Netherlands; Department of Cardiology, University Medical Centre Utrecht, Utrecht, The Netherlands; Department of Medical Informatics, Academic Medical Centre, University of Amsterdam, Amsterdam, The Netherlands

**Keywords:** Cardiac output, Oxygen uptake, Patterns, Chronic heart failure, Exercise intolerance

## Abstract

**Background:**

Patients with chronic heart failure (CHF) suffer from exercise intolerance due to impaired central hemodynamics and subsequent alterations in peripheral skeletal muscle function and structure. The relative contribution of central versus peripheral factors in the reduced exercise capacity is still subject of debate. The main purpose was to investigate heterogeneity in the nature of exercise intolerance by evaluating individual cardiac output (Q) patterns. The secondary purpose was to evaluate whether patient and disease characteristics were associated with a central hemodynamic exercise limitation.

**Methods:**

Sixty-four stable CHF patients performed a symptom limited incremental exercise test with respiratory gas analysis and simultaneous assessment of Q, using a radial artery pulse contour analysis method. A central hemodynamic exercise limitation was defined as a plateau or decline in Q from 90 to 100 % of exercise duration.

**Results:**

Data from 61 patients were analyzed. A central hemodynamic exercise limitation was observed in 21 patients (34 %). In these patients, a higher occurrence of a plateau/decrease in oxygen uptake (VO_2_) (52 % vs 23 %, p = 0.02), stroke volume (SV) (100 % vs. 75 %, *p* = 0.01) and chronotropic incompetence (31 % vs. 2.5 %, *p* = 0.01) was observed, while presence of a left bundle branch block (LBBB) occurred significantly less (19 % vs 48 %, p = 0.03) There was no difference in disease characteristics such as etiology, duration, NYHA class, mitral regurgitation or ischemia.

**Conclusions:**

The study revealed considerable heterogeneity in the nature of exercise limitations between moderately impaired CHF patients. In one third of the study population a plateau or decrease in Q towards peak exercise was demonstrated, which is indicative of a central hemodynamic exercise limitation. A central hemodynamic exercise limitation was associated with an impairment to augment stroke volume and heart rate.

## Background

Chronic heart failure (CHF) is a clinical syndrome resulting from inadequate tissue oxygenation due to impaired cardiac function. Although it is well established that patients with CHF suffer from exercise intolerance, the underlying pathophysiological mechanisms remain controversial. From a theoretical point of view, reduced exercise capacity may be the consequence of O_2_ diffusion abnormalities in the lungs, impaired central hemodynamics or peripheral derangements such as a reduced skeletal muscle capillarization or metabolic capacity. Whereas it is generally accepted that pulmonary O_2_ diffusion does not limit exercise capacity in compensated CHF patients, the role of central and peripheral factors is still under debate [1]. Previous studies showed associations between maximal exercise capacity and central hemodynamics [2, 3], whereas other studies revealed a relation between exercise capacity and skeletal muscle function [4, 5]. A possible explanation for these seemingly discrepant findings is that the nature of exercise limitations differs between CHF patients. In fact, results from an animal study demonstrated that the primary response of microvascular oxygen pressure in the muscle was speeded in rats with moderate left ventricular (LV) dysfunction, while in rats with severe LV dysfunction, this response was significantly slowed [6]. These results suggest that the dynamic balance between oxygen delivery and utilization in the muscle is dependent of the severity of cardiac dysfunction, indicating physiological heterogeneity. Although additional physiological insight is needed in humans, some clinical studies suggested heterogeneity in exercise limitations. As such, CHF patients with a severe central hemodynamic limitation did not benefit from exercise training [7], but may benefit most from a heart transplantation [8]. Characterization of exercise limitations in individual CHF patients and more knowledge on disease characteristics associated with the nature of these limitations may lead to a more tailored therapeutic strategy in CHF patients. An approach that can be used to characterize exercise limitations is to examine the pattern of the cardiac output (Q) response to symptom-limited exercise, with a failure to augment or a decrease in cardiac output towards peak exercise being indicative of a central hemodynamic exercise limitation [9]. Although previous studies investigated the cardiac output response to exercise with respect to prognosis [10, 11], no studies used cardiac output patterns during incremental exercise to characterize exercise limitations in CHF patients.

The primary goal of this study was to investigate the nature of the limitation of maximal exercise capacity in CHF patients by evaluation of cardiac output patterns. Furthermore we evaluated whether patient and disease characteristics are associated with a central hemodynamic exercise limitation.

## Methods

The present study was designed as a prospective cohort study. All tests were conducted at the Department of Cardiology of the Máxima Medical Centre, the Netherlands. The research protocol was approved by the medical ethics committee of the Máxima Medical Centre. The study complies with the Declaration of Helsinki. All patients provided written and signed informed consent, prior to the study.

### Population

All consecutive symptomatic CHF patients visiting the outpatient clinic of cardiology were considered for participation in the study. Additional inclusion criteria were: CHF secondary to ischemic or dilated cardiomyopathy, New York Heart Association functional class II-III, left ventricular ejection fraction ≤ 40 % and optimized medical treatment. Exclusion criteria were: recent myocardial infarction, unstable angina (less than 3 months prior to inclusion), hemodynamically significant aortic valve disease, participation in a training program (≥2/week) in the last year, significant chronic obstructive pulmonary disease (FEV1/FVC < 60 %) and orthopedic or neuromuscular conditions limiting the ability to perform exercise.

### Exercise testing

All patients performed a symptom-limited incremental exercise test on an electromagnetically braked cycle ergometer in an upright position (Corrival, Lode, Groningen, The Netherlands), using an individualized ramp protocol with a duration of 8 to 12 min. The test ended when a patient was not able to maintain the required pedaling frequency of 70 per minute. A 12-lead electrocardiogram was registered continuously. Ventilatory parameters were measured breath-by-breath (ZAN 680 USB, ZAN Messgeräte, Oberthulba, Germany). Volume and gas analyzers were calibrated before each test.

### Assessment of central hemodynamics

Assessment of central hemodynamics was performed by a radial artery pulse contour analysis method (LiDCO, LiDCO Ltd, London, UK). This technique provides beat-to-beat changes in central hemodynamics, by calculating nominal stroke volume (SV) from a pressure-volume transform of the radial artery pressure waveform [12]. In order to convert nominal SV to absolute SV, the system has to be calibrated at rest for each subject by an independent method. We used echocardiography or lithium dilution for this purpose [13, 14]. Before the exercise test, a 20-gauge arterial catheter was inserted into the radial artery. The radial artery catheter was connected to the LiDCO *plus* monitor. Subsequently, the calibration procedure to determine resting Q was performed in the supine position. Thereafter patients were positioned upright on the cycle ergometer and the exercise protocol was started. Patients were instructed to keep the measured arm on the handlebar of the ergometer in the same position to keep an optimal arterial waveform. Previous studies showed that LiDCO is a reproducible and accurate method for assessment of cardiac output (Q) under a variety of physiological conditions [15, 16]. Moreover, in a study using the Fick method as a reference, we showed that this technique is highly accurate for continuous assessment of Q during incremental symptom-limited exercise testing in CHF patients [13].

### Data analysis

Breath-to-breath data of oxygen uptake and beat-to-beat data of central hemodynamics were filtered for outliers using Python (Python 2.7, Python Software Foundation). Outliers were defined as values deviating more than three standard deviations from a calculated moving average [17]. All data were time aligned using manual set markers at the start of exercise and filtered using a central moving average filter with a window of 11 data points. In order to compare gas exchange and central hemodynamic variables during exercise between CHF patients, data were expressed as a percentage of total exercise time. Baseline values were calculated as the mean of the first 60 s of the unloaded phase prior to the start of loaded exercise. Peak values were defined as the average values during the final 20 s of the test. We characterized exercise limitations by using the pattern of the cardiac output response to maximal exercise. A failure to increase or a decrease in cardiac output towards the end of exercise was considered to be indicative of a central hemodynamic exercise limitation. A central hemodynamic exercise limitation was defined as a plateau or decline in Q from 90 to 100 % of exercise time. Chronotropic incompetence was defined as a peak HR below 80 % of the age-predicted heart rate, using the Brawner formula [18].

### Statistical analysis

Data were analyzed using SPSS 19.0.0 statistical software (SPSS Inc, Chicago, IL, USA). Continuous variables were tested for normality and presented as mean or mean ± SD. Between-group differences of continuous variables were evaluated by an independent *t*-test. Categorical variables are presented as absolute and relative frequencies. The *χ*2 test was used to evaluate differences between categorical data. Differences were presented as *χ*2 value with concomitant degree of freedom (df) Relations between variables were assessed by Pearson’s correlation coefficient (*r*). For all statistical comparisons, the level of significance was set at *p* < 0.05.

## Results

All 64 patients completed the combined hemodynamic and cardiopulmonary exercise test, without adverse events. Central hemodynamic data from 3 patients were excluded because of insufficient data quality due to excessive damping of the arterial pressure waveform during exercise. In total, data of 61 patients were analyzed.

The majority of the study population were males (84 %); the mean age was 63 ± 9 years. At the time of inclusion, patients were diagnosed with CHF for a mean duration of 45 ± 51 months. Ninety-eight percent used ACE inhibitors or an angiotensin II receptor blocker, 93 % used beta blockers. Thirty-four patients had an ischemic cardiomyopathy (due to one or more myocardial infarctions > 3 months prior to inclusion), 2 patients showed signs of myocardial ischemia during evaluation by non-invasive stress testing (i.e. positive exercise test or myocardial perfusion scintigraphy) or coronary angiography, which required percutaneous coronary intervention (PCI). Six patients had severe mitral regurgitation at resting echocardiography. Twenty-three patients had Left Bundle Branch Block (LBBB) and seven patients had atrial fibrillation.

### Central hemodynamic and gas exchange variables at rest and maximal exercise

Gas exchange and hemodynamic variables at rest and peak exercise are presented in Table 1. The mean peak workload was 125 ± 49 Watt. The mean peak VO_2_ was 19.0 ± 5.9 mL min^−1^ kg^−1^ and VO_2_ at the ventilatory threshold was 11.9 ± 2.9 mL min^−1^ kg^−1^, corresponding to 63 % of peak VO_2_. Mean peak RER was 1.07 ± 0.1. There was a significant correlation between peak VO_2_ and peak Q (*r* =0.64, *p* < 0.001).

**Table 1:** Hemodynamic and gas exchange variables at rest and during exercise (n =61)

	Baseline	Peak exercise
VO_2_ (mL min^−1^ kg^−1^)	4.3 ± 1.3	19.0 ± 5.9
HR (beats min^−1^)	80 ± 17	124 ± 26
SV (mL)	62 ± 14	87 ± 23
Q (L min^−1^)	4.9 ± 1.5	10.9 ± 4.1

VO_2_ oxygen uptake, HR heart rate, SV stroke volume, Q Cardiac Output

### Patterns of central hemodynamic variables during incremental exercise

**Fig. 1:**
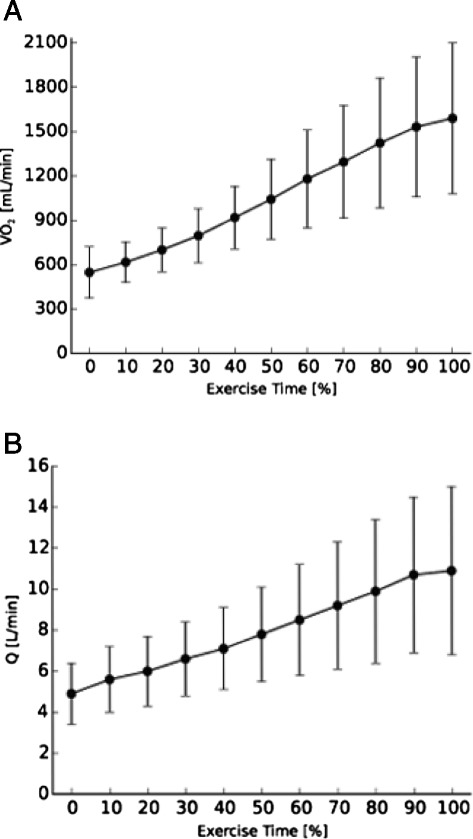
**a**: Mean oxygen uptake (VO_2_) and **b**: mean cardiac output (Q) response during a symptom limited exercise test. Error bars represent one standard deviation above and below the mean

**Fig. 2:**
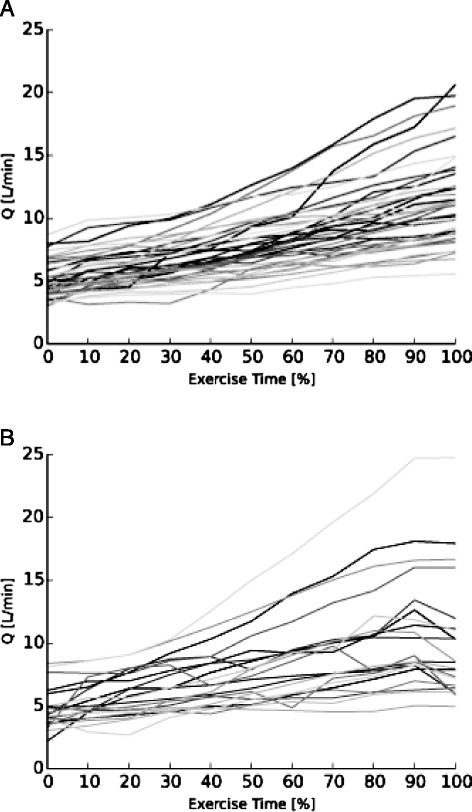
**a**: Individual cardiac output (Q) responses with an continuous increase during exercise (*n* =40) and **b**: individual cardiac output (Q) responses with a plateau or decrease during the final 10 % of exercise during a symptom limited exercise test (*n* =21)

Fig. 1a and b show respectively, exercise-induced changes of VO_2_ and Q at a group level. Both VO_2_ and Q show a continuous increase throughout the exercise test. Looking at patterns of Q in individual patients, 21 patients (34 %) showed a plateau or decrease in Q during the final 10 % of exercise duration (Fig. 2b), while 40 patients (66 %) showed a continuous increase in Q (Fig. 2a).

Table 2 shows a comparison of patient and disease characteristics between patients with a plateau/decrease in Q and patients with a continuous increase in Q. There was no significant between-group difference in beta blocker use (*χ*2 value 0.17, df = 1, *p* =0.68). Peak VO_2_, peak Q and SV did not differ between both groups. In 11 of the 21 patients with a plateau/decrease in Q (52 %), a plateau/decrease in VO_2_ was also observed. Nine out of 40 patients (23 %) with a continuous increase in Q, showed a plateau/decrease in VO_2_ (*χ*2 value 5.58, df = 1, *p* = 0.02 for between group comparison). In patients with a plateau/decrease in Q, all patients showed a plateau/decrease in SV as opposed to 30 patients (75 %) in the group with a continuous increase in Q (*χ*2 value 6.28, df = 1, *p* = 0.01 for between group comparison) (Table 2). Chronotropic incompetence was observed more often in patients with a plateau or decrease in Q (31 % versus 2.5 %, *χ*2 value 7.05, df = 1, p < 0.01). There was a higher occurrence of a plateau/decrease in SV in patients with chronotropic incompetence (83 % versus 52 %, *p* =0.047), but no significant difference in peak SV between patients with and without chronotropic incompetence (74 ± 22 versus 89 ± 22 mL respectively, *p* =0.29). Left Bundle Branch Block was observed significantly less in patients with a Q plateau/decrease (*χ*2 value 4.84, df = 1, p = 0.03). No differences between groups were observed for patients with severe mitral regurgitation at rest or myocardial ischemia.

**Table 2:** Characteristics comparison between subjects with increase in Q or plateau/decrease in Q (n = 61)

	Increase in Q (*n* = 40)	Plateau/decrease in Q (*n* = 21)	*χ*2 value (df)	*p-value*
Age (years)	63 ± 10	64 ± 9	n.a.	NS
Gender male/female (%)	33 (83)/7 (17)	18 (86)/3 (14)	0.10 (1)	NS
Etiology ICM/DCM (%)	21 (53)/19 (47)	13 (62)/8 (38)	0.49 (1)	NS
Duration CHF (months)	45 ± 50	46 ± 54	n.a.	NS
Beta blocker (%)	93	95	0.17 (1)	NS
NYHA class II/III (%)	24 (60)/16 (40)	11 (52)/10 (48)	0.33 (2)	NS
LVEF (%)	31 ± 8	33 ± 10	n.a.	NS
Peak VO_2_ (mL min^−1^ kg^−1^)	19.4 ± 6.0	18.2 ± 5.9	n.a.	NS
Peak Q (L min^−1^)	11.3 ± 3.6	10.2 ± 5.0	n.a.	NS
Peak SV (mL)	90 ± 24	83 ± 22	n.a.	NS
Plateau/decrease in VO_2_	9 (23 %)	11 (52 %)	5.58 (1)	0.02
Plateau/decrease in SV	30 (75 %)	21 (100 %)	6.28 (1)	0.01
Rest HR (beats min^−1^)	81 ± 16	77 ± 18	n.a.	NS
Peak HR (beats min^−1^)	126 ± 20	121 ± 35	n.a.	NS
Chronotropic incompetence	1 (2.5 %)	5 (31 %)	7.05 (1)	0.01
Rhythm (SR, Afib, paced) (%)	35 (88)/2 (5)/3 (7)	15 (71)/5 (24)/1 (5)	4.85 (2)	NS
LBBB	19 (48 %)	4 (19 %)	4.84 (1)	0.03
Severe mitral regurgitation	3 (7.5 %)	3 (14 %)	0.72 (1)	NS
Myocardial ischemia	2 (5 %)	0 (0 %)	1.09 (2)	NS

*χ*2 value, chi-squared value for categorical values, df degree of freedom,n.a. not applicable, ICM ischemic cardiomyopathy, DCM dilated cardiomyopathy, CHF chronic heart failure, NYHA New York Heart Association, LVEF left ventricular ejection fraction, VO_2_ oxygen uptake, Q cardiac output, SV stroke volume, HR heart rate, SR sinus rhythm, Afib atrial fibrillation, LBBB left bundle branch block, . NS non significant

## Discussion

This is the first study in patients with CHF to characterize physiological limitations at maximal exercise by assessment of the pattern of Q during incremental exercise. In one third of the study population we demonstrated a failure to augment Q towards the end of the exercise test, (Fig. 2b) while a continuous increase was observed in the other patients (Fig. 2a). These results indicate that physiological heterogeneity in exercise limitations exists among CHF patients.

As the nature of the exercise limitation may be an important determinant for the selection of patients for specific treatments, (e.g. exercise training, heart transplantation, cardiac resynchronization therapy) these observations may be relevant for clinical practice.

Although we demonstrated a continuous increase in Q throughout the incremental symptom-limited exercise test at group level, we observed a wide inter-individual variation in Q patterns. These observations are different from earlier findings in healthy individuals, showing a continuous increase in Q in all subjects [19]. Whereas individual Q patterns during symptom-limited exercise were not used previously to characterize exercise limitations in CHF patients, earlier studies did show heterogeneity in central hemodynamic responses to exercise in CHF populations by relating Q to VO_2_. In these studies, 32-55 % of CHF patients were classified as having a central hemodynamic limitation of maximal exercise capacity [7, 8], which is in line with our study. Studies using other methods to investigate determinants of maximal exercise capacity in CHF patients demonstrated strong relations between maximal exercise capacity and both skeletal muscle function, e.g. reduced skeletal muscle metabolic capacity or peripheral O_2_ transport [4, 5, 20], as well as central hemodynamics [2, 3]. However, these results did not allow to draw conclusions on the relative contributions of central and peripheral factors to impaired maximal exercise capacity, nor on the presence of physiological heterogeneity in CHF patients. Studies investigating the influence of peripheral and central factors on exercise capacity simultaneously are scarce and yielded conflicting results. Whereas some of these studies indicate that intrinsic differences in skeletal muscle metabolism are the main determinants of a reduced exercise capacity [21, 22], other studies show that a reduced O_2_ delivery to exercising muscles is the primary limiting factor [23, 24]. Possible explanations for this discrepancy may be the variety in exercise protocols that were used but also physiological heterogeneity in exercise limitations in these study populations.

One third of our population showed a central hemodynamic limitation of maximal exercise capacity. This limitation was associated with a more frequent occurrence of a plateau/ decrease in VO_2_. Furthermore a central exercise limitation was associated both with a higher occurrence of a plateau/decrease in SV and a higher occurrence of chronotropic incompetence. From a physiological point of view, the inability to augment forward SV may be caused by impaired global left atrial [25] or ventricular contractile reserve, regional contractility disorders due to ischemia, valvular disorders such as mitral regurgitation and dyssynchrony. As severe mitral regurgitation at rest and ischemia were not identified as determinants of a central exercise limitation, an impaired global contractile reserve is likely to be the most important determinant of impaired SV augmentation in our population. In addition to a failure to augment SV, a central exercise limitation was also associated with a higher occurrence of chronotropic incompetence . In theory, this observation may be caused by neurohormonal dysregulation or the use of beta blockers. Previous studies showed that the lower peak heart rate during long-term administration of beta blockers is associated with a higher peak SV [26, 27]. In contrast, in our study population, patients with chronotropic incompetence, had a higher occurrence of failure to augment SV and a non-statistically significant lower peak SV. A previous study in CHF patients showed a significant correlation between peak oxygen uptake and change in heart rate, with no significant difference for patients with or without beta blockers [28]. As 93 % patients in our study used beta blockers and there was no difference in beta blocker use in both groups, this could not explain the difference in occurrence of chronotropic incompetence. Therefore, we postulate that chronotropic incompetence based on neurohormonal dysregulation (i.e. an imbalance between sympathic and parasympathic nerve activity) can be a contributory factor limiting exercise capacity in patients with a central exercise limitation. Our results showed significantly less patients with LBBB in the centrally limited group. This could be explained by studies that provide evidence that left ventricular (i.e. mechanical) dyssynchrony is irrespective of the QRS duration. Moreover, it is suggested that dyssynchrony is a dynamic condition and may worsen during exercise [29].

Characterization of exercise limitations may be beneficial in clinical practice for a better understanding of the causes of impairments in daily functioning. In addition, this approach may contribute to a more tailored therapeutic strategy in individual CHF patients. As such, Wilson et al. showed that patients with a reduced cardiac output response to exercise benefit less from a moderate intensity exercise training program than patients with a normal cardiac output response [7]. Yet, other studies showed that patients with a more pronounced central exercise limitation may benefit more from interventions aimed at improving central hemodynamics such as heart transplantation [8] and cardiac resynchronization therapy [30]. Whether the approach used in the present study will contribute to a better prediction of treatment results in individual CHF remains to be determined.

Before drawing conclusion from this study, several limitations should be acknowledged. First, we used an arbitrary method to classify exercise limitations. In fact, some patients that were not classified as having a central exercise limitation showed only small increases in Q during the final part of exercise. Second, the limited sample size did not permit to perform additional subgroup analyses. For instance, due to the fact that most patients were moderately impaired, we were unable to test the hypothesis, generated from animal studies that patient with more severe left ventricular dysfunction at rest have a more pronounced peripheral limitation than those with moderate left ventricular dysfunction [6]. Third, assessment of valvular disease and dyssynchrony was performed by resting echocardiography. However, as both may worsen during exercise [31], we cannot fully exclude that this factor played a role in the failure to augment SV and Q towards maximal exercise.

## Conclusion

This study revealed heterogeneity in exercise limitations in CHF patients. In one third of the study population a plateau or decrease in Q towards peak exercise was demonstrated, which is indicative of a central exercise limitation. Factors associated with a central exercise limitation included a higher occurrence of a failure to augment SV and chronotropic incompetence, suggesting that both impaired contractile reserve and neurohormonal dysregulation are determinants of reduced exercise capacity in centrally limited patients. Future research should focus on the clinical utility of characterizing exercise limitations to predict treatment effects in CHF patients.

## References

[1] Cohen-Solal A, Logeart D, Guiti C, Dahan M, Gourgon R (1999). Cardiac and peripheral responses to exercise in patients with chronic heart failure. Eur Heart J.

[2] Hummel YM, Bugatti S, Damman K, Willemsen S, Hartog JWL, Metra M, Sipkens JS, van Veldhuisen DJ, Voors AA (2012). Functional and hemodynamic cardiac determinants of exercise capacity in patients with systolic heart failure. Am J Cardiol.

[3] Sullivan MJ, Knight JD, Higginbotham MB, Cobb FR (1989). Relation between central and peripheral hemodynamics during exercise in patients with chronic heart failure. Muscle blood flow is reduced with maintenance of arterial perfusion pressure. Circulation.

[4] Okita K, Yonezawa K, Nishijima H, Hanada A, Ohtsubo M, Kohya T, Murakami T, Kitabatake A (1998). Skeletal Muscle Metabolism Limits Exercise Capacity in Patients With Chronic Heart Failure. Circulation.

[5] Hopkinson NS, Dayer MJ, Antoine-Jonville S, Swallow EB, Porcher R, Vazir A, Poole-Wilson P, Polkey MI (2013). Central and peripheral quadriceps fatigue in congestive heart failure. Int J Cardiol.

[6] Diederich ER, Behnke BJ, McDonough P, Kindig CA, Barstow TJ, Poole DC, Musch TI (2002). Dynamics of microvascular oxygen partial pressure in contracting skeletal muscle of rats with chronic heart failure. Cardiovasc Res.

[7] Wilson JR, Groves J, Rayos G (1996). Circulatory status and response to cardiac rehabilitation in patients with heart failure. Circulation.

[8] Chomsky DB, Lang CC, Rayos GH, Shyr Y, Yeoh TK, Pierson RN, Davis SF, Wilson JR (1996). Hemodynamic exercise testing. A valuable tool in the selection of cardiac transplantation candidates. Circulation.

[9] Critoph CH, Patel V, Mist B, Elliott PM (2014). Cardiac output response and peripheral oxygen extraction during exercise among symptomatic hypertrophic cardiomyopathy patients with and without left ventricular outflow tract obstruction. Heart.

[10] Myers J, Wong M, Adhikarla C, Boga M, Challa S, Abella J, Ashley EA (2013). Cardiopulmonary and noninvasive hemodynamic responses to exercise predict outcomes in heart failure. J Card Fail.

[11] Lang CC, Karlin P, Haythe J, Lim TK, Mancini DM (2009). Peak cardiac power output, measured noninvasively, is a powerful predictor of outcome in chronic heart failure. Circ Heart Fail.

[12] Jonas MM, Tanser SJ (2002). Lithium dilution measurement of cardiac output and arterial pulse waveform analysis: an indicator dilution calibrated beat-by-beat system for continuous estimation of cardiac output. Curr Opin Crit Care.

[13] Kemps HMC, Thijssen EJM, Schep G, Sleutjes BTHM, De Vries WR, Hoogeveen AR, Wijn PFF, Doevendans PAFM (2008). Evaluation of two methods for continuous cardiac output assessment during exercise in chronic heart failure patients. J Appl Physiol.

[14] Lang RM, Bierig M, Devereux RB, Flachskampf FA, Foster E, Pellikka PA, Picard MH, Roman MJ, Seward J, Shanewise J, Solomon S, Spencer KT, St. John Sutton M, Stewart W: Recommendations for chamber quantification. Eur J Echocardiogr 2006;7:79–108. 10.1016/j.euje.2005.12.014 16458610

[15] Hamilton TT, Huber LM, Jessen ME (2002). PulseCO: a less-invasive method to monitor cardiac output from arterial pressure after cardiac surgery. Ann Thorac Surg.

[16] Linton NW, Linton RA (2001). Estimation of changes in cardiac output from the arterial blood pressure waveform in the upper limb. Br J Anaesth.

[17] Lamarra N, Whipp B, Ward S, Wasserman K (1987). Effect of interbreath fluctuations on characterizing exercise gas exchange kinetics. J Appl Physiol.

[18] Brubaker PH, Kitzman DW (2011). Chronotropic incompetence: causes, consequences, and management. Circulation.

[19] Higginbotham MB, Morris KG, Williams RS, McHale PA, Coleman RE, Cobb FR (1986). Regulation of stroke volume during submaximal and maximal upright exercise in normal man. Circ Res.

[20] Esposito F, Mathieu-Costello O, Shabetai R, Wagner PD, Richardson RS (2010). Limited maximal exercise capacity in patients with chronic heart failure: partitioning the contributors. J Am Coll Cardiol.

[21] Shoemaker JK, Naylor HL, Hogeman CS, Sinoway LI (1999). Blood Flow Dynamics in Heart Failure. Circulation.

[22] Wiener DH, Fink LI, Maris J, Jones RA, Chance B, Wilson JR (1986). Abnormal skeletal muscle bioenergetics during exercise in patients with heart failure: role of reduced muscle blood flow. Circulation.

[23] Toussaint JF, Koelling TM, Schmidt CJ, Kwong KK, LaRaia PJ, Kantor HL (1998). Local relation between oxidative metabolism and perfusion in leg muscles of patients with heart failure studied by magnetic resonance imaging and spectroscopy. J Heart Lung Transplant.

[24] Sperandio PA, Borghi-Silva A, Barroco A, Nery LE, Almeida DR, Neder JA (2009). Microvascular oxygen delivery-to-utilization mismatch at the onset of heavy-intensity exercise in optimally treated patients with CHF. Am J Physiol Heart Circ Physiol.

[25] Ceresa M, Capomolla S, Pinna GD, Febo O, Caporotondi A, Guazzotti GP, La Rovere MT, Francolini G, Olivares A, Gnemmi M, Mortara A, Maestri R, Cobelli F (2002). Left atrial function: bridge to central and hormonal determinants of exercise capacity in patients with chronic heart failure. Monaldi Arch Chest Dis.

[26] Metra M, Nardi M, Giubbini R, Dei Cas L (1994). Effects of short- and long-term carvedilol administration on rest and exercise hemodynamic variables, exercise capacity and clinical conditions in patients with idiopathic dilated cardiomyopathy. J Am Coll Cardiol.

[27] Eynon N, Sagiv M, Amir O, Ben-Sira D, Goldhammer E, Amir R. The effect of long-term beta-adrenergic receptor blockade on the oxygen delivery and extraction relationship in patients with coronary artery disease. J Cardiopulm Rehabil Prev, 2008;28:189–94. 10.1097/01.HCR.0000320070.81470.75 18496318

[28] Magrì D, Palermo P, Cauti FM, Contini M, Farina S, Cattadori G, Apostolo A, Salvioni E, Magini A, Vignati C, Alimento M, Sciomer S, Bussotti M, Agostoni P (2012). Chronotropic Incompentence and Functional Capacity in Chronic Heart Failure: No Role of β-Blockers and β-Blocker Dose. Cardiovasc Ther.

[29] Lancellotti P, Moonen M (2012). Left ventricular dyssynchrony: a dynamic condition. Heart Fail Rev.

[30] Schlosshan D, Barker D, Pepper C, Williams G, Morley C, Tan LB (2006). CRT improves the exercise capacity and functional reserve of the failing heart through enhancing the cardiac flow- and pressure-generating capacity. Eur J Heart Fail.

[31] Lapu-bula R, Robert A, Craeynest D Van, Dhondt A, Gerber BL, Pasquet A, Melin JA, Kock M De. Contribution of Exercise-Induced Mitral Regurgitation to Exercise Stroke Volume and Exercise Capacity in Patients. Circulation 2002;106(11):1342–8. 10.1161/01.cir.0000028812.98083.d9 12221050

